# Pyruvate Protects Pathogenic Spirochetes from H_2_O_2_ Killing

**DOI:** 10.1371/journal.pone.0084625

**Published:** 2014-01-02

**Authors:** Bryan Troxell, Jun-Jie Zhang, Travis J. Bourret, Melody Yue Zeng, Janice Blum, Frank Gherardini, Hosni M. Hassan, X. Frank Yang

**Affiliations:** 1 Department of Microbiology and Immunology, Indiana University School of Medicine, Indianapolis, Indiana, United States of America; 2 Department of Biology, University of Nebraska Kearney, Nebraska, United States of America; 3 Department of Microbiology, North Carolina State University, Raleigh, North Carolina, United States of America; 4 Laboratory of Zoonotic Pathogens, National Institute of Allergy and Infectious Diseases, National Institute of Health, Hamilton, United States of America; University of Toledo School of Medicine, United States of America

## Abstract

Pathogenic spirochetes cause clinically relevant diseases in humans and animals, such as Lyme disease and leptospirosis. The causative agent of Lyme disease, *Borrelia burgdorferi*, and the causative agent of leptospirosis, *Leptospria interrogans*, encounter reactive oxygen species (ROS) during their enzootic cycles. This report demonstrated that physiologically relevant concentrations of pyruvate, a potent H_2_O_2_ scavenger, and provided passive protection to *B. burgdorferi* and *L. interrogans* against H_2_O_2_. When extracellular pyruvate was absent, both spirochetes were sensitive to a low dose of H_2_O_2_ (≈0.6 µM per h) generated by glucose oxidase (GOX). Despite encoding a functional catalase, *L*. *interrogans* was more sensitive than *B. burgdorferi* to H_2_O_2_ generated by GOX, which may be due to the inherent resistance of *B. burgdorferi* because of the virtual absence of intracellular iron. In *B. burgdorferi*, the nucleotide excision repair (NER) and the DNA mismatch repair (MMR) pathways were important for survival during H_2_O_2_ challenge since deletion of the *uvrB* or the *mutS* genes enhanced its sensitivity to H_2_O_2_ killing; however, the presence of pyruvate fully protected Δ*uvrB* and Δ*mutS* from H_2_O_2_ killing further demonstrating the importance of pyruvate in protection. These findings demonstrated that pyruvate, in addition to its classical role in central carbon metabolism, serves as an important H_2_O_2_ scavenger for pathogenic spirochetes. Furthermore, pyruvate reduced ROS generated by human neutrophils in response to the Toll-like receptor 2 (TLR2) agonist zymosan. In addition, pyruvate reduced neutrophil-derived ROS in response to *B. burgdorferi*, which also activates host expression through TLR2 signaling. Thus, pathogenic spirochetes may exploit the metabolite pyruvate, present in blood and tissues, to survive H_2_O_2_ generated by the host antibacterial response generated during infection.

## Introduction

Lyme disease, the most common vector born disease in the United States, is a chronic, system wide infection caused by the pathogen *Borrelia burgdorferi* (*B. burgdorferi*) [1]–[3]. *B. burgdorferi* transmission occurs from its arthropod tick vector, *Ixodes* sp., during feeding on its human host. *Ixodes* ticks have a usual two-year life cycle that includes three distinct stages: larvae; nymph; and adult. At each developmental stage, ticks will feed once on a warm-blooded host and undergo a molting process that may proceed for a period of months. Small rodents (especially the white-footed mouse, *Peromyscus leucopus*) are the primary reservoirs for *B. burgdorferi* and sources for the bloodmeal during the larvael and nymphal stages of the tick vector. *P. leucopus* mice infected with *B. burgdorferi* exhibit minimal tissue pathology and no protective immune response, which demonstrates the susceptibility of this host to chronic infection. In order to survive in both hosts, *B. burgdorferi* must be able to overcome multiple host defense mechanisms, one of which is bacteriocidal reactive oxygen species (ROS) produced by host cells from both ticks and mammals. In this regard, it has been shown that tick salivary proteins provide significant protection to *B. burgdorferi* against ROS at the site of tick feeding [4]–[8]. How *B. burgdorferi* defends against H_2_O_2_ after dissemination to systemic sites during mammalian infection remains largely unknown.

As an obligated parasite, *B. burgdorferi* has a reduced genome and limited enzymes that defend against host ROS attack. *B. burgdorferi* encodes a superoxide dismutase (Mn-SOD) that supports rapid conversion of superoxide anion radical (O_2_^−^) to hydrogen peroxide (H_2_O_2_) [9], [10], but lacks genes encoding catalase or hydroperoxidase that defend against H_2_O_2_. This predicts that, in general, *Borrelia* spp. are inept at defending against H_2_O_2_. Although the NADH-dependent Coenzyme A disulphide reductase (CoADR) has been implicated in H_2_O_2_ defense [11], deletion of this gene (*bb0728*) does not influence H_2_O_2_ sensitivity [12]. Reports have been conflicting with respect to the sensitivity of *B. burgdorferi* to H_2_O_2_. Some suggest that *B. burgdorferi* is resistant to H_2_O_2_ killing [13], [14], perhaps due to low intracellular iron that prevents DNA damage due to the Fenton reaction [15]. Yet other studies report that *B. burgdorferi* is sensitive to H_2_O_2_ [16], [17]. Thus, the response of *B. burgdorferi* to H_2_O_2_ remains controversial.

Leptospirosis is a multisystem disease caused by *Leptospira interrogans* (*L. interrogans*) and exists in nature within a zoonotic cycle. Similar to the enzootic cycle of *B. burgdorferi*, small rodents, especially rats, are considered critical hosts in the spread of *L. interrogans* since rats can shed the pathogen in urine and exist as asymptomatic carriers [18]. *L. interrogans* encodes a functional catalase that is homologous to the heme-dependent KatE family [19], [20]. During interaction with host innate immune cells, transcription of *katE* is enhanced suggesting an importance during infection [21]. Recent work demonstrates the importance of *katE* during infection of hamsters [22] implying that H_2_O_2_ is a bacteriocidal component of the host defense against *L. interrogans*.

Pyruvate is a fundamental compound within central carbon metabolism. For over a century, it also has been known that pyruvate can be oxidatively decarboxylated by H_2_O_2_ to produce CO_2_ and acetate [23], [24]. In fact, when peroxide is present it interferes with enzymatic assays that utilize pyruvate [25]. Therefore, pyruvate and other related metabolites have been shown to protect the cell from ionizing radiation, a stress that generates ROS and H_2_O_2_ *in vitro* [24]–[31]. Furthermore, pyruvate can ameliorate the pathological inflammation associated with kidney failure *in vivo* [32]. Pyruvate is a common additive to the medium for cultivation of eukaryotic cells. During *in vitro* cultivation cells are exposed to higher O_2_ concentrations and greater H_2_O_2_ stress than that *in vivo* (21% O_2_ *in vitro* versus ≈4–7% O_2_ *in vivo*) [33], [34], Indeed, the importance of pyruvate in the cultivation medium is independent of its role as a carbon source, but is due to its potent H_2_O_2_-scavenging ability [30], [35]. Given that the standard Barbour-Stoenner-Kelly (BSK-II) medium for cultivation of *B. burgdorferi* contains 7 mM pyruvate and that the addition of pyruvate to the cultivation medium of *L. interrogans* enhances cultivation [36], pyruvate present in the medium may serve a beneficial role by scavenging H_2_O_2_ that accumulates in complex growth media [37], [38]. Moreover, pyruvate may account for the conflicting results in previous reports on the sensitivity of *B. burgdorferi* to H_2_O_2_.

This study demonstrates that *B. burgdorferi* and *L. interrogans* were sensitive to a low dose of H_2_O_2_ killing generated by the enzyme glucose oxidase (GOX); however, both pathogens were protected by physiologically relevant concentrations of extracellular pyruvate. The Lyme disease spirochete requires DNA repair pathways to repair H_2_O_2_-damaged DNA since mutants within the nucleotide excision repair (NER) and the DNA mismatch repair (MMR) pathways exhibited enhanced sensitivity to H_2_O_2_ killing; however, the presence of pyruvate fully protected Δ*uvrB* and Δ*mutS* from H_2_O_2_ killing further demonstrating the important role of pyruvate in survival. Exogenous pyruvate reduced the ROS production by peripheral blood human neutrophils upon stimulation with the Toll-like receptor 2 (TL2) agonists, *B. burgdorferi* or zymosan. These results demonstrate that the ubiquitous metabolite, pyruvate, which is present in blood and other tissues, had a previously unappreciated role in protecting the extracellular pathogens *B. burgdorferi* and *L. interrogans* from H_2_O_2_ stress and may protect these pathogens during their enzootic cycles.

## Materials and Methods

### Bacterial Strains, Growth Conditions, and Reagents

*B. burgdorferi* and *L. interrogans* strains used throughout are listed below. Infectious strain 5A4NP1 is a B31-A3 derived strain (a gift from Drs. H. Kawabata and S. Norris, University of Texas Health Science Center at Houston), which contains a kanamycin resistant gene that disrupts the restriction modification gene *bbe02* located on linear plasmid 25 (lp25) [39]. Strain AH130 is an infectious 297 derived strain recovered from an infected patient [40], which has been passed once through laboratory mice [41], [42]. Infectious strain B31-A3 is a clonal isolate from B31-MI [43], which was originally isolated from *Ixodes dammini* ticks on Shelter Island, New York, USA [44].

*B. burgdorferi* were grown in BSK medium prepared and supplemented with 6% heat inactivated rabbit serum as described previously ([45], BSK-II) at 37°C in a 5% CO_2_ incubator. *L. interrogans* strains were cultivated in liquid Ellinghausen-McCullough-Johnson-Harris (EMJH) medium at 30°C [46], [47].

Sodium pyruvate, H_2_O_2_, streptomycin, and kanamycin sulfate (used at 300 µg ml^−1^) were purchased from Fisher Scientific. Glucose oxidase (E.C. 1.1.3.4, β-D-glucose:oxygen 1-oxidoreductase, GOX), bovine catalase (E.C. 1.11.1.6, H_2_O_2_:H_2_O_2_ oxidoreductase), and N-acetyl cysteine (NAC) were purchased from Sigma-Aldrich (St. Louis, MO).

### Generation of mutS and uvrB Deletions in B. burgdorferi

The *mutS* gene (*bb0797*) was deleted from *B. burgdorferi* strain B31-A3 by homologous recombination with the suicide vector pPCR-Script Cam::*ΔmutS::aadA* (pCm::*ΔmutS-aadA*) using a strategy described previously [48]. Briefly, PCR products encoding 1 kb regions upstream and downstream of *mutS* were amplified from DNA isolated from *B. burgdorferi* strain B31-A3 with La *Taq* polymerase (Takara Bio, Madison, WI), while the *aadA* streptomycin resistance cassette under the control of a *flgB* promoter (*flgBP-aadA*) was amplified from the vector pKFSS1 [49] using primers encoding restriction sites for downstream cloning (**Table 1**) into pPCR-Script CamSK(+) (Agilent Technologies, Cedar Creek, TX). The PCR products were directionally cloned into pPCR-Script Cam SK(+) using restriction enzymes and T4 DNA ligase (New England Biolabs, Ipswich, MA). Electrocompetent *B. burgdorferi* B31-A3 cells [50] were transformed with 40 µg of pCm::*ΔmutS-aadA* and *mutS* mutants were selected for by plating on BSK-II plating medium supplemented with 40 µg ml^−1^ streptomycin. Deletion of *mutS* was verified by PCR using primers listed in **Table 1**.

**Table 1:** Primers used in this study.

Primer name	Sequence (5′-3′)	Reference
mutS-KpnI-F	ACTGTAGGTACCTGTAAATACTGTGCAGATATC	This study
mutS-EcoRI-R	ACTGTAGAATTCTCTTGATATCTAAATACTGC	This study
mutS-EcoRI-F	ACTGTAGAATTCTCAGTCCAGTTAGTATGAGC	This study
mutS-SacI-R	ACTGTAGAGCTCATTCCTGGAGTAGAAAGCTC	This study
aadA-EcoRI-F	ACTGTAGAATTCTACCCGAGCTTCAAGGAA	This study
aadA-EcoRI-R	ACTGTAGAATTCTATTTGCCGACTACCTTG	This study

### H_2_O_2_ Killing Assays

Strains 5A4NP1 and AH130 were grown in BSK-II medium to late log phase. Five×10^7^ cells ml^−1^ were centrifuged at 10,000×*g* for 10 minutes and thrice washed with 0.9% NaCl. Samples were resuspended to 5×10^7^ cells ml^−1^ in either BSK-II or modified BSK-II that did not contain pyruvate. Bacteria were challenged with 1 or 2.5 mM H_2_O_2_ for 1 hour at 37°C in a 5% CO_2_ incubator. The reaction was stopped by the addition of 1,000 U ml^−1^ of bovine catalase and bacteria were centrifuged as above. Cell pellets were washed twice in BSK-II medium containing 1,000 U ml^−1^ of catalase and resuspended in 1 ml of BSK-II medium. Quantification of viability was determined by limiting dilution in 96-well plates (Fisher Scientific, Pittsburgh, PA) following incubation at 37°C/5% CO_2_ for 14 days [51]. Controls were incubated in BSK-II and BSK-II without pyruvate and treated with catalase for comparison with H_2_O_2_ treated samples.

Alternatively, the viability of wild-type *B. burgdorferi* B31-A3 and the isogenic *ΔmutS* and *ΔuvrB* strains following challenge with H_2_O_2_ in the presence or absence of pyruvate was determined by enumerating colony-forming units (CFU) on BSK-II plates. Briefly, strains were grown in BSK-II medium under microaerobic conditions 3% O_2_, 5% CO_2_ at 34°C to late log phase. Cell cultures were pelleted by centrifugation at 1200 g for 10 min, washed twice in HN buffer (20 mM NaCl, 50 mM Hepes, pH 7.6) and resuspended in HN buffer +0.2% glucose to a cell density of ≈5×10^7^ cells ml^−1^. One ml aliquots of cells were transferred to 5 ml polypropylene culture tubes, followed by the addition of sodium pyruvate and/or H_2_O_2_. Cultures were incubated at 34°C for 2 h followed by serial dilution in HN buffer and plating on BSK-II. Plates were incubated under microaerobic conditions 3% O_2_, 5% CO_2_ at 34°C for 7–14 days to allow for enumeration of CFU. Percent survival for each strain was calculated by dividing the CFU from plates with treated samples by the CFU from plates with untreated samples.

### GOX Treatment of Spirochetes

To determine H_2_O_2_ production by GOX in BSK-II medium, 0.32 mU ml^−1^ of GOX was added to sterile BSK-II containing or lacking pyruvate and incubated at 37°C/5% CO_2_. H_2_O_2_ was measured using a commercially available kit ([52] National Diagnostics, Atlanta, Georgia, USA) for 100 h at 37°C/5% CO_2_. Samples of H_2_O_2_ in BSK-II media were diluted 5-fold prior to quantification of H_2_O_2_ [52]. A standard curve of (see below) H_2_O_2_ was run in parallel. In sterile BSK-II medium without pyruvate, the rate of H_2_O_2_ production was linear for ≈24 h and maintained the concentration of H_2_O_2_ above 10 µM for 100 h.

To determine the influence of pyruvate on GOX activity in sterile BSK-II medium, 10 units of GOX was added to BSK-II that contained and lacked pyruvate. Samples were incubated as above and the concentration of glucose was measured for 6 h using a commercially available kit (R-BioPharm, Marshall, Michigan, USA).

Strains 5A4NP1 and AH130 were grown as above and inoculated into BSK-II medium containing or lacking pyruvate (7 mM) to 10^5^ cells ml^−1^. A concentrated preparation of GOX, in 50 mM sodium acetate buffer pH 5.1, was diluted in BSK-II to a final concentration of 0.32 mU ml^−1^. Growth of spirochetes was monitored by quantification using dark field microscopy over time. Subsequent experiments utilized a relevant concentration of pyruvate, 100 µM. *L. interrogans* serovars Manilae L495 and Lai 56601 [46], [47] were inoculated to 10^6^ spirochetes ml^−1^ in EMJH medium plus glucose (2 g L^−1^) with or without sodium pyruvate (910 µM). Medium without added glucose was used as a control. After 1.5 days of growth, GOX was added to the medium at a final concentration of 0.32 mU ml^−1^. Growth was monitored by counting spirochetes by dark-field microscopy. Three independent experiments were performed with two or three replicates for each treatment.

### Reaction of H_2_O_2_ with Pyruvate

The kinetics of the reaction of H_2_O_2_ with pyruvate was determined as follows. A sample of H_2_O_2_ was prepared, in 50 mM phosphate buffer pH 7.0, by determining the concentration of a 100-fold dilution of a concentrated stock (Fisher Scientific, Pittsburgh, PA). The absorbance at 240 nm was determined and the concentration was determined using a molar extinction coefficient of 43.6 M^−1^ cm^−1^ [53]. A standard curve of 1–100 µM H_2_O_2_ was prepared and run in parallel. One mM of H_2_O_2_ from this solution was added to media that contained or lacked pyruvate at 7 mM and samples were removed and H_2_O_2_ was quantified as above.

The reciprocal version of this experiment was performed by measuring the concentration of pyruvate using a commercially available kit (Abcam, Cambridge, Massachusetts, USA). BSK-II medium was prepared without added pyruvate then 7 mM H_2_O_2_ was added. One mM of pyruvate was added, samples were removed over time and added to a sample of catalase to stop the reaction. A standard of sodium pyruvate was prepared as previously described by measuring absorbance at 316 nm with an extinction coefficient of 18 M^−1^ cm^−1^ [54]. A standard curve of pyruvate was quantitated in parallel with unknown samples.

To calculate the half-life (t_1/2_) of the reaction in BSK-II medium, the concentration of pyruvate was an excess of 7-fold compared to H_2_O_2_ (pseudo-first order reaction). The concentration of H_2_O_2_ was measured over time as above. A plot of Ln[H_2_O_2_] *versus* time revealed a straight line within the first 5 minutes of the reaction; the negative slope of this line is equal to the rate constant (*k*). Data from the linear portion of this graph (0 to 5 minutes) were used to calculate the t_1/2_ using the equation t_1/2_ = Ln2/*k*. The same experiment was performed with the concentration of H_2_O_2_ present in excess of 7-fold of pyruvate. Pyruvate was measured over time as above and the t_1/2_ was determined as above.

### Production of Acetate from the Reaction of Pyruvate with H_2_O_2_

A solution of H_2_O_2_ was prepared as above and 1 mM was added to BSK-II medium. Over time, aliquots were removed and added to tubes containing 200 U ml^−1^ of bovine catalase to stop the reaction. The concentration of acetate produced from the reaction was determined using a commercially available kit from R-Biopharm. To confirm the reaction was pyruvate-dependent, H_2_O_2_ was added to BSK-II medium that lacked pyruvate. To confirm that H_2_O_2_ is required, H_2_O_2_ was added to BSK-II medium that contained bovine catalase. Also, in a separate experiment, the concentration of pyruvate was manipulated in BSK-II medium that was prepared without the addition of pyruvate. Bovine catalase was used to stop the reactions that contained different equimolar concentrations of pyruvate/H_2_O_2_ and the concentration of acetate was determined as above.

### Stimulation of Superoxide Production in Human Neutrophils by *B. burgdorferi*

This study was approved by Indiana University School of Medicine Institute Review Boards, and written informed consent was obtained from donors. Human neutrophils were isolated from heparin-anticoagulated venous blood using Polymorphprep (AXIS-SHIELD PoC, Oslo, Norwary). Superoxide production by neutrophils stimulated with live or heat-inactivated *B. burgdorferi,* with or without pyruvate, was measured by luminol chemiluminescence assay [55]. Specifically, 2.5×10^5^ human neutrophils were plated into a well of a 96-well plate (COSTAR, Corning, NY) in PBSG (PBS plus 0.9 mM CaCl_2_, 0.5 mM MgCl_2_, 20 mM dextrose) in the presence of 50 µM luminol and horseradish peroxidase (HRP; final concentration: 20 U ml^−1^), without or with superoxide dismutase (SOD; final concentration: 75 µg ml^−1^) and kept on ice for 10 minutes prior to the assay. *B. burgdorferi* (2.5×10^6^ cells) in 25 µl of PBS were added to wells, and the plate was spun down at 800 r.p.m. for 1 min immediately prior to reading. Relative light units (RLU) were monitored at 60-second intervals for up to one hour by the Long Kinetic module in an Lmax microplate luminometer (Molecular Devices, Sunnyvale, CA). Integrated RLU values were calculated by SOFTmax software (Molecular Devices).

### Statistical Analyses

Statistical significance was determined using a Student’s *t* test and when multiple comparisons were made the *p* value was corrected using the Bonferroni correction. When comparing three or more groups to a control value, One way Analysis of Variance (ANOVA) was used. Because data expressed as percent impart a fixed range on the data (0–100%), the percent values were transformed to arcsine values using the formula SIN^−1(√(%/100))^ prior to One way ANOVA testing. A post-hoc test (Dunnett’s multiple comparisons) was used to test significance. Some experiments used a Two-way ANOVA to determine significance.

## Results

### Pyruvate Protects *Borrelia burgdorferi* against H_2_O_2_

*B. burgdorferi* encounters ROS stress during its infectious cycle. H_2_O_2_ diffuses across prokaryotic and eukaryotic cell membranes with the permeability much like H_2_O to impair cellular functions [56]–[60]. Conflicting results have been reported on the response of *B. burgdorferi* to H_2_O_2_ [13], [14], [16], [17]. We noticed that an important distinction among these reports is the difference in methodology; when *B. burgdorferi* is challenged with H_2_O_2_ in BSK-II medium it is highly resistant [13], [14], but when challenged in minimal buffers it is sensitive [16], [17]. We hypothesized that pyruvate (7 mM) present in BSK-II medium may account for the previous observations of H_2_O_2_ resistance [13], [14], [16].

**Figure 1:**
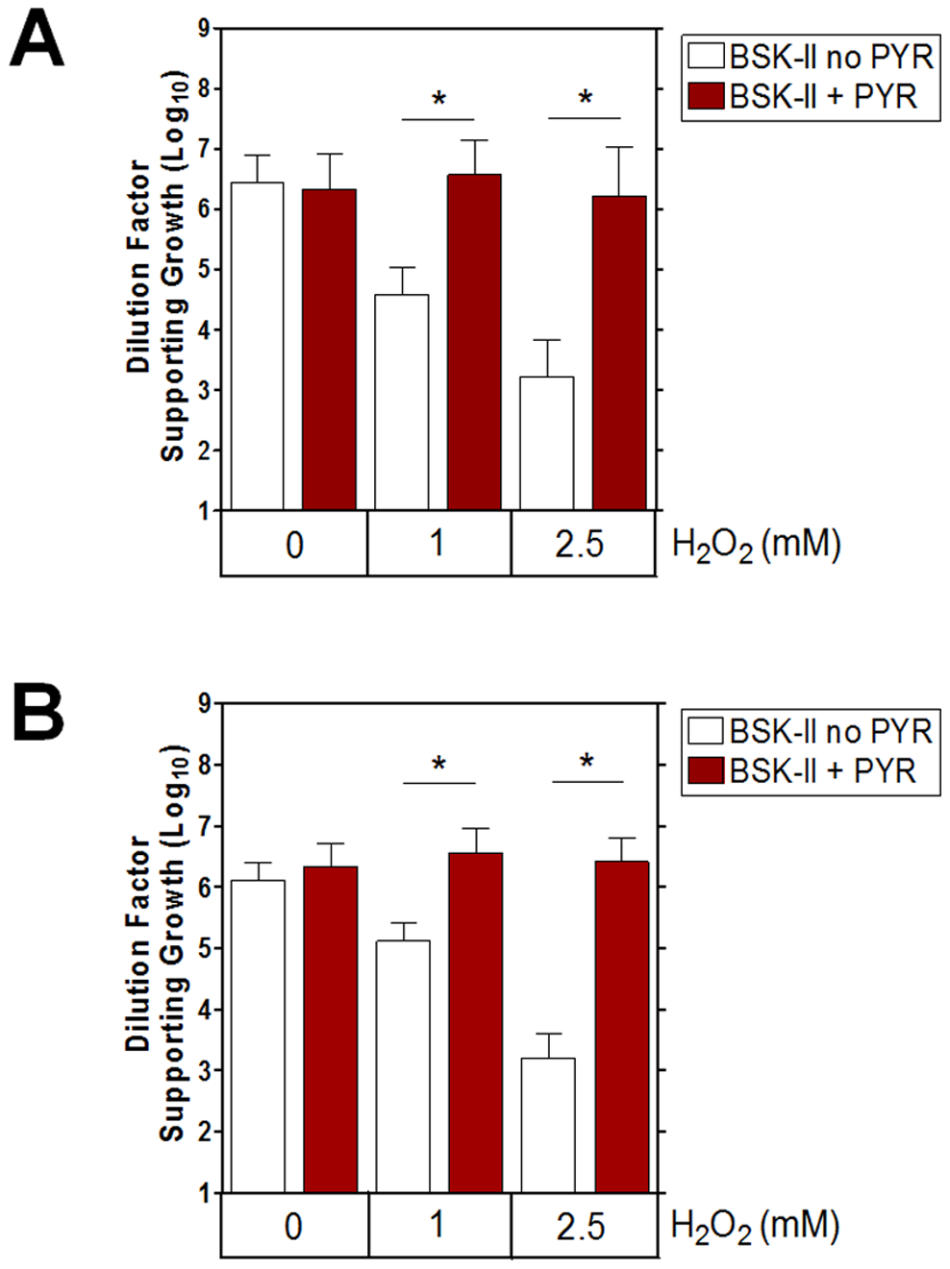
Pyruvate Protects *Borrelia burgdorferi* From H_2_O_2_ Stress. (**A**) 5A4NP1 and (**B**) AH130 were challenged in BSK-II medium for 1 h with various concentrations of H_2_O_2_ in the presence or absence of pyruvate. Viability was determined by limiting dilution. Data shown are mean +/− SD from three separate experiments. A paired Student’s t test determined significance (* p≤0.025, corrected by Bonferroni adjustment).

To test the hypothesis that the presence of pyruvate in BSK-II medium protects against H_2_O_2_, we compared spirochete killing by H_2_O_2_ in either standard BSK-II medium or in BSK-II medium which lacked pyruvate. Two infectious strains of *B. burgdorferi*, 5A4NP1 (an isolate of strain B31) and AH130 (an isolate of strain 297), were incubated with various concentrations of H_2_O_2_ for one hour and cell viability was assessed by limiting dilution method. Similar to the work of others [13],[14], when cultured in BSK-II media with pyruvate the spirochetes were resistant to H_2_O_2_ treatment (**Fig. 1**). However, when pyruvate was excluded from the medium, 1 mM H_2_O_2_ was sufficient to reduce spirochete viability by over 1 log, and spirochete killing by H_2_O_2_ was dose dependent (**Fig. 1AB**). Thus, we conclude that pyruvate passively protects *B. burgdorferi* from H_2_O_2_.

**Figure 2:**
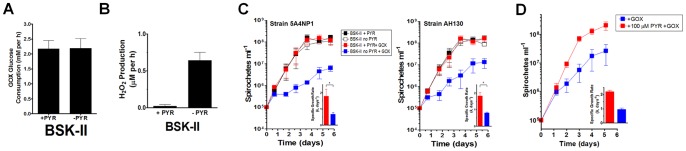
*B. burgdorferi* Are Sensitive to a Low Dose of H_2_O_2_ Produced by Glucose Oxidase (GOX). (**A**) Pyruvate does not alter GOX activity in BSK-II medium. Ten units of GOX were added to BSK-II medium containing or lacking pyruvate. Glucose was measured for 6 h to determine GOX activity. Data shown are mean +/− SD (n = 2). (**B**) The presence of pyruvate reduces the presence of H_2_O_2_ in BSK-II medium. GOX (0.32 mU ml^−1^) was added to BSK-II medium containing or lacking pyruvate and H_2_O_2_ was measured for 100 h. The linear rate of H_2_O_2_ production over 24 h is shown. Data are from 2 separate experiments performed in 2 separate batches of medium. (**C**) 5A4NP1 (**left panel**) and AH130 (**right panel**) were inoculated in BSK-II medium containing or lacking pyruvate in the presence or absence of GOX. Data shown are mean +/− SD from three separate experiments. The inset shows the specific growth rate (*k*, days^−1^) during cultivation in GOX-containing BSK-II with (red bar) and without (blue) pyruvate. GOX was added at 0.32 mU ml^−1^. A paired Student’s t test determined significance (* p≤0.05). (**D**) Experiments from (**C**) were repeated with strain 5A4NP1 using a physiologically relevant concentration of sodium pyruvate (100 µM) with GOX treatment. Data shown are from two separate experiments and the inset shows the specific growth rate (*k*, days^−1^) during cultivation in GOX-containing BSK-II with (red bar) and without (blue) pyruvate. GOX was added at 0.32 mU ml^−1^.

Experiments from **Fig. 1** demonstrate the protective role of pyruvate in preserving borrelial viability during exposure to a single pulse of H_2_O_2_. Although such an approach is commonly used to assess H_2_O_2_ sensitivity for many cell types, this experimental design does not recapitulate the scenario of H_2_O_2_ stress *in vivo* where pathogens often encounter low doses of sustained H_2_O_2_ production [61]. For instance, the *ex vivo* rate of H_2_O_2_ produced by activated macrophages has been measured at ≈0.15 nmol h^−1^ per 10^5^ macrophages [62]. To expose *B. burgdorferi* to a steady, low dose concentration of H_2_O_2_, glucose oxidase (GOX) from *Aspergillus niger* was used. This flavoenzyme allowed steady production of H_2_O_2_ and D-glucono-δ-lactone from glucose and O_2_. GOX activity was not influenced by pyruvate, as the presence or absence of pyruvate had no influence on the glucose consumption by GOX in BSK-II medium (**Fig. 2A**). The presence of 0.32 mU ml^−1^ of GOX in BSK-II medium (lacking pyruvate) was sufficient to generate a rate of H_2_O_2_ at 0.62±0.1 µM per h (or 0.62 nmoles ml^−1^) (**Fig. 2B**) and maintained the concentration of H_2_O_2_ above 10 µM for 100 h (**data not shown**). The addition of pyruvate to BSK-II medium significantly reduced the detection of H_2_O_2_. Thus, such a system (BSK-II minus pyruvate plus GOX) allowed us to determine the prolonged impact of steady low levels of H_2_O_2_ on the growth of *B. burgdorferi.* When cultivated within such medium, *B. burgdorferi* exhibited a significant growth defect (**Fig. 2C**). Pyruvate is a ubiquitous metabolite. In human blood, pyruvate is typically within a range of ≈50 to 300 µM, but is subject to significant flux [63]–[67]. BSK-II medium contains 7 mM pyruvate, which is substantially higher than concentrations of pyruvate *in vivo*. Therefore, to determine the relevance of the protection against H_2_O_2_ by pyruvate, we repeated the experiments shown in **Fig. 2C** with a physiologically relevant concentration of pyruvate, 100 µM. Using strain 5A4NP1, it is apparent that 100 µM protects against a low dose of H_2_O_2_ generated by GOX (**Fig. 2D**). These results indicate that *B. burgdorferi* is sensitive to the physiological relevant concentration of H_2_O_2_ and that a relevant concentration of pyruvate allows uninhibited growth in the presence of H_2_O_2_.

### Pyruvate Protects *B. burgdorferi* DNA Repair Mutants from Oxidative Stress

**Figure 3:**
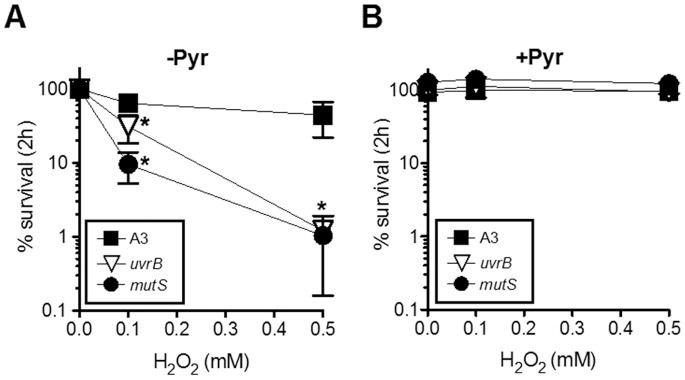
Pyruvate Protects DNA Repair Mutants Strains From ROS. Wild-type *B. burgdorferi* B31 A3 and the isogenic DNA repair mutants *ΔuvrB* and *ΔmutS* were exposured to 0, 0.1 or 0.5 mM H_2_O_2_ in HN buffer +0.2% glucose in the (**A**) absence or (**B**) presence of 2.5 mM pyruvate for 2 h. Percent survival was calculated by enumerating CFU. Data represent the mean +/− SD (n = 4). Two-way ANOVA was used to determine significance (* p<0.01).

Because of *B. burgdorferi*’s lack of intracellular iron that could participate in the Fenton reaction, *B. burgdorferi* was predicted to be resistant to oxidative DNA damage when treated with H_2_O_2_ [15]. This prediction was supported by the observation that *B. burgdorferi* challenged with high concentrations of H_2_O_2_ were resistant to killing and did not show evidence of DNA damage [13]. However, recent studies have shown that *B. burgdorferi* strains harboring mutations in NER genes (*uvrA, uvrB* and *uvrC*) are hypersensitive to killing and DNA damage following exposure to either ROS or RNS [48], [68], [69]. These observations suggest that the lack of DNA damage in ROS-challenged wild-type cells is likely due to efficient DNA repair. To determine whether exogenous pyruvate could protect *B. burgdorferi* from H_2_O_2_-mediated DNA damage, we compared the sensitivity of strain B31-A3 and isogenic mutants in the NER gene *uvrB* and the MMR gene *mutS* to increasing concentrations of H_2_O_2_ in HN buffer supplemented with 0.2% glucose. Both the *uvrB* and *mutS*-deficient mutant displayed significant reductions in survival compared to wild-type cells when challenged with either 100 or 500 µM H_2_O_2_ (**Fig. 3A**). The addition of 500 µM H_2_O_2_ reduced the survival of wild-type cells to 44% following 2 h of exposure, while only 1% of *uvrB* or *mutS*-deficient cells survived. This supports a crucial role for both the NER and MMR pathways in the repair of oxidative DNA damage. Despite the hypersensitivity of the *uvrB* or *mutS*-deficient strains to oxidative stress, the addition of 2.5 mM pyruvate completely protected both from the lethal effects of 100 and 500 µM H_2_O_2_ (**Fig. 3B**). Collectively, these data support the H_2_O_2_-scavenging role for pyruvate and demonstrate the necessity of both the NER and MMR pathways for protection of the *B. burgdorferi* genome from potential lethal oxidative DNA lesions.

### Pyruvate Reacts Directly with H_2_O_2_

**Figure 4:**
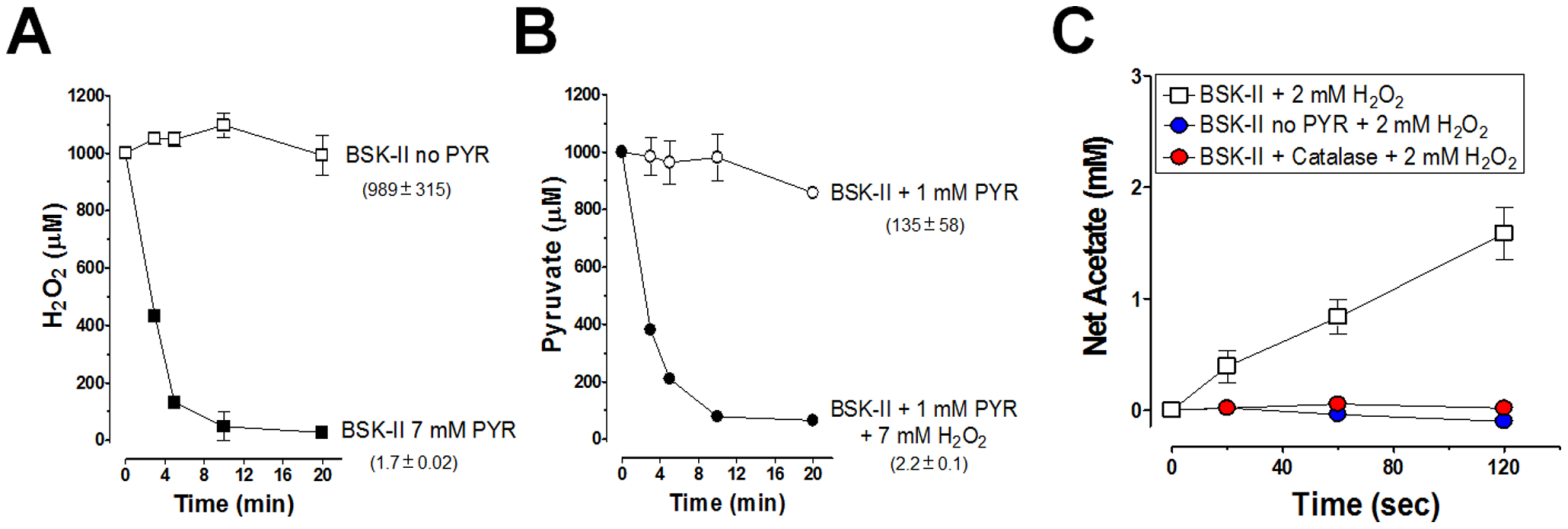
Pyruvate and H_2_O_2_ Produce Acetate. (**A**) One mM H_2_O_2_ was added to pyruvate-free BSK-II and standard BSK-II and H_2_O_2_ was measured over time using the xylenol orange/ferric iron assay [52]. (**B**) One mM pyruvate was added to pyruvate-free BSK-II. H_2_O_2_ (7 mM) was added and pyruvate was measured over time using a commerically available kit. Data shown are mean +/− SD (n = 3). The half-life values, in minutes, are shown in parentheses. (**C**) Production of acetate by pyruvate and H_2_O_2_ requires both reactants in BSK-II medium. The production of acetate was measured over time in BSK-II medium that contained pyruvate (7 mM), lacked pyruvate, and contained pyruvate with the addition of bovine catalase (200 U ml^−1^).

BSK-II medium is a complex medium that contains 6% rabbit serum, 5% BSA, and other ingredients. To determine whether pyruvate in BSK-II protects *B. burgdorferi* by scavenging H_2_O_2_ and if so, how fast the reaction between H_2_O_2_ and pyruvate occurs in BSK-II, and whether other ingredients of BSK-II significantly react with H_2_O_2_, we measured the degradation of 1 mM of H_2_O_2_ in BSK-II medium containing or lacking 7 mM pyruvate. The result showed that the half-life of H_2_O_2_ was 1.7 minutes in BSK-II, but 989 minutes in BSK-II medium lacking pyruvate, a difference of 580-fold (**Fig. 4A**). This result indicates that pyruvate is a primary factor that promotes the breakdown of H_2_O_2_ in BSK-II medium. We also measured the consumption of pyruvate in BSK-II medium containing and lacking 7 mM H_2_O_2_, and the result confirmed the reaction between pyruvate and H_2_O_2_ (**Fig. 4B**).

The reaction between pyruvate and H_2_O_2_ generates CO_2_ and acetate [24]. To confirm this reaction occurred in BSK-II medium, acetate production was measured over time. The production of acetate occurred when both pyruvate and H_2_O_2_ were present in BSK-II medium (**Fig. 4C**). Furthermore, the production of acetate was blocked by catalase, indicating the dependence of this reaction on H_2_O_2_ (**Fig. 4C**). Thus, pyruvate protects *B. burgdorferi* against H_2_O_2_ by a non-enzymatic reaction between pyruvate and H_2_O_2_ to produce acetate (and CO_2_).

### Pyruvate Protects Pathogenic *Leptospira interrogans* against H_2_O_2_ Killing

**Figure 5:**
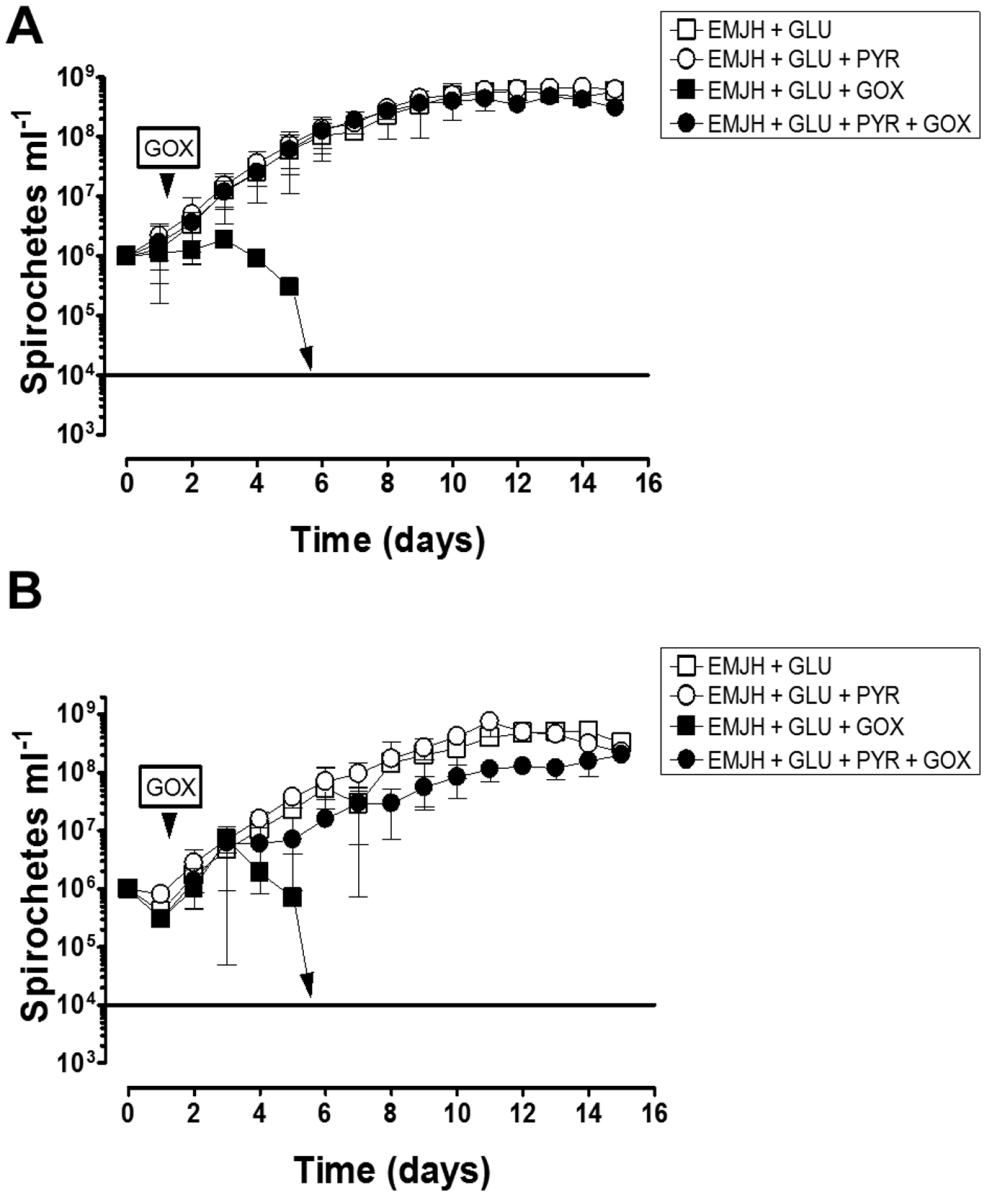
Pyruvate Protects Pathogenic Strains of *Leptospira interrogans* Against H_2_O_2_. (**A**) *L. interrogans* serovar Manilae L495 was challenged with GOX (0.32 mU ml^−1^) in EMJH medium with and without added pyruvate (PYR). Stationary phase cultures were diluted to 10^6^ cells ml^−1^ in EMJH medium with glucose (2 g L^−1^) and with or without PYR (910 µM). After 1.5 days of growth, GOX was added, indicated by the arrowhead and growth was monitored by direct counting of spirochetes by dark field microscopy. The limit of detection (LOD) for direct counting is indicated by a solid line and cultures with spirochetes below the LOD are indicated by the arrow pointing to the line, which corresponds to ≈10^4^ spirochetes ml^−1^. (**B**) *L. interrogans* serovar Lai 56601 was challenged with GOX as described above. Results in (**A**) and (**B**) show the mean ±1 SD from three separate experiments performed in duplicate or triplicate.

Because pathogenic spirochetes exist mainly as extracellular pathogens *in vivo* and pyruvate exists in extracellular fluids *in vivo*, it is possible that spirochetes other than *B. burgdorferi* are protected against H_2_O_2_ with relevant concentrations of pyruvate. To test this, two pathogenic strains of *L. interrogans* serovar Manilae L495 (**Fig. 5A**) and serovar Lai 56601 (**Fig. 5B**) were tested for growth defects in the presence of GOX with and without added pyruvate. Strains were cultivated in EMJH medium with added glucose (GLU) with or without added pyruvate. As with *B. burgdorferi*, the addition of pyruvate did not influence the growth of either *L. interrogans* serovar in liquid cultures (**Fig. 5AB**). Following the addition of GOX to cultures that lacked added pyruvate, the concentration of serovars Manilae L495 and serovar Lai 56601 spirochetes decreased appreciably until day 6 when no detectable spirochetes were present (limit of detection is 10^4^ spirochetes ml^−1^, **Fig. 5AB**). This demonstrates a decrease of ≥3 orders of magnitude of spirochete cell density from days 3 to 6. However, the presence of pyruvate protected both pathogenic strains from GOX challenge (**Fig. 5AB**). Despite the presence of catalase within these pathogenic strains, both were sensitive to a low dose of H_2_O_2_ generated by GOX, which was abrogated by the addition of pyruvate to the culture medium. Thus, pyruvate protects both catalase positive (*L. interrogans*) and catalase negative (*B. burgdorferi*) spirochetes from H_2_O_2_ killing.

### Pyruvate Reduces Reactive Oxygen Species Exposure of *B. burgdorferi* by Human Neutrophils

**Figure 6:**
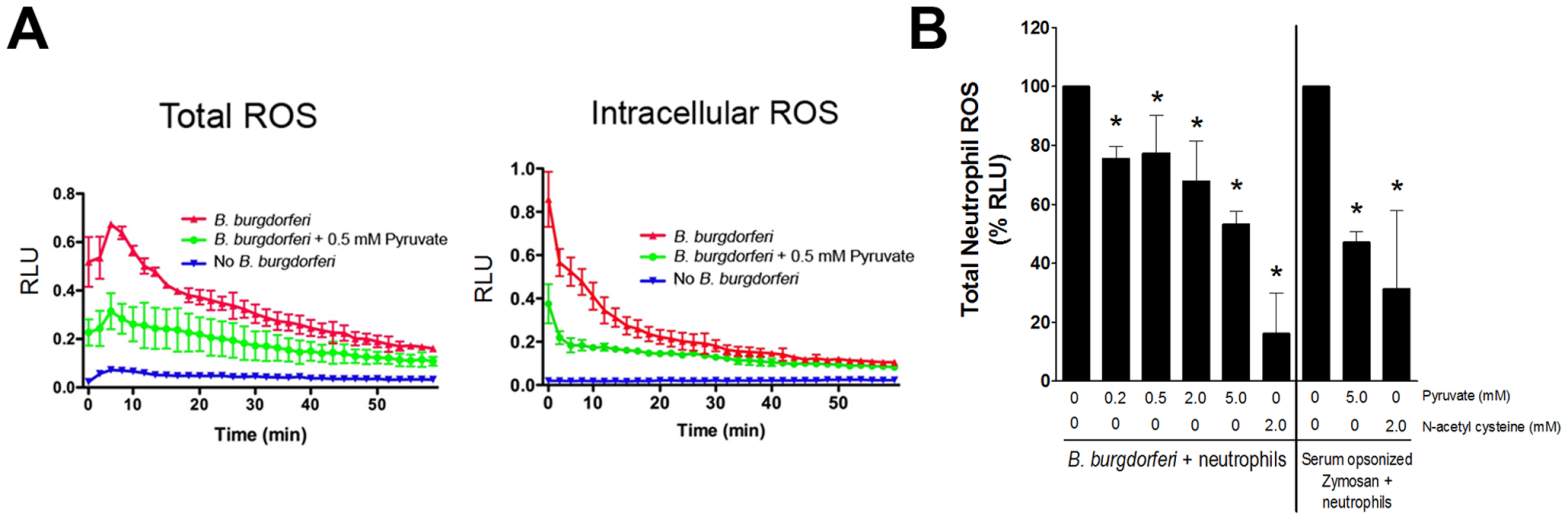
Pyruvate Reduces the Detection of ROS Generated by Human Neutrophils. (**A**) Human peripheral neutrophils were incubated with *B. burgdorferi* 5A4NP1 strain, ratio of 10∶1 *B. burgdorferi*:neutrophil, and ROS generation over 1 h was determined using the luminol detection assay and are expressed as Relative Light Units (RLU). (**B**) Human peripheral neutrophils were incubated with 5A4NP1, ratio of 10∶1 *B. burgdorferi*:neutrophil, and ROS generation over 1 h was determined using the luminol detection assay. Data shown are the integrated RLU for the duration of 3 separate experiments. Zymosan (SOZ) is a positive control for neutrophil activation and N-acetylcysteine (NAC) is a positive control for reduction of ROS. ROS produced by neutrophils with *B. burgdorferi* was set to 100% and the significance was compared to neutrophils+*B. burgdorferi*+antioxidant (pyruvate or NAC) using One-way ANOVA. Although percentages are shown, data were transformed to arcsine values using the formula SIN^−1(√(%/100))^ prior to One way ANOVA testing (* p<0.05). ROS produced by neutrophils with SOZ was set to 100% and significance was determined with One way ANOVA (* p<0.05) compared to the same condition with added antioxidant.

The above results demonstrate that pyruvate protects *B. burgdorferi* from H_2_O_2_ killing under *in vitro* cultivation conditions. Given that during infection, myeloid derived immune cells are potent generators of H_2_O_2_, it is unknown whether pyruvate would react with ROS produced by neutrophils in response to *B. burgdorferi* stimulus. Neutrophils were chosen to test this since they are recruited to sites of *B. burgdorferi* infection [70], [71] and are known to generate cytotoxic molecules capable of killing *B. burgdorferi* [17], [72]. Neutrophils from peripheral human blood were isolated, incubated with *B. burgdorferi*, and ROS detection was determined in the presence or absence of pyruvate using a luminol-based assay [55], [73]. Peripheral neutrophils elicit robust ROS production in response to *B. burgdorferi* (**Fig. 6A**). However, the presence of 0.5 mM pyruvate dramatically reduced ROS production by human neutrophils (**Fig. 6A**). Neutrophils are capable of localizing the NADPH oxidase complex to the plasma membrane [74]. Thus, we expected to see a decrease in the extracellular ROS produced by neutrophils in the presence of pyruvate. Surprisingly, intracellular ROS was also reduced by pyruvate, suggesting that pyruvate may enter neutrophil phagosomes. To characterize further the influence of pyruvate on ROS production by human neutrophils, we repeated these experiments with different concentrations of pyruvate. Results are presented as percentage of integrated RLU [55]. As a control, we used the antioxidant N-acetyl-L-cysteine (NAC), which is a known scavenger of ROS and inhibitor of mitogen-activated protein kinase (MAPK) activation of the NADPH oxidase complex [75], [76]. A potent activator of ROS production by neutrophils, serum opsonized zymosan (SOZ), through TLR2 served as the positive control [77], [78]. ROS produced by neutrophils and *B. burgdorferi* was set to 100% and compared to neutrophils+*B. burgdorferi*+antioxidant (either pyruvate or NAC). The addition of 0.2 mM of pyruvate significantly reduced the ROS production of *B. burgdorferi*-stimulated neutrophils by >20% compared to neutrophils stimulated in the absence of pyruvate (**Fig. 6B**). Furthermore, 5 mM pyruvate decreased the production of ROS by neutrophils stimulated *B. burgdorferi* by ≈50% (**Fig. 6B**). Although 0.2, 0.5, and 2 mM significantly reduced the total ROS production by neutrophils compared to no addition of pyruvate, the difference of inhibition was not significant when comparing between 0.2, 0.5, and 2 mM pyruvate. This suggests that the pyruvate-dependent decrease in production of ROS by neutrophils may exhibit a bimodal pattern of inhibition. For SOZ controls, pyruvate was capable of decreasing the ROS produced by the positive control SOZ by ≈50%. For this comparison, neutrophils+SOZ was set to 100%. Thus, pyruvate is a strong scavenger of ROS in the context of neutrophil activation by *B. burgdorferi* or SOZ. As expected, NAC reduced the ROS produced by *B. burgdorferi* stimulated neutrophils by ≈80% and by ≈65% for SOZ stimulated neutrophils (**Fig. 6B**). These data suggest that pyruvate reduces the exposure of *B. burgdorferi* to ROS produced by neutrophils.

## Discussion

*B. burgdorferi* lacks classical enzymatic defenses against H_2_O_2_, leaving this spirochete as shown here, susceptible to killing by this membrane permeable form of ROS (**Figs. 1** **, 2CD, and 3**). Yet, contradictory reports regarding *B. burgdorferi* sensitivity to H_2_O_2_ have to this point remained unexplained [13], [14], [16], [17]. The current study revealed an essential role for pyruvate in protecting *B. burgdorferi* from H_2_O_2_ toxicity. Despite the plethora of data demonstrating the protective role of pyruvate and related 2-oxo acids against H_2_O_2_ [23]–[32], the ability of pyruvate to protect against H_2_O_2_ killing in the context of pathogenic bacteria has been overlooked. In the complex medium of BSK-II without pyruvate, H_2_O_2_ slowly dissipated, but when pyruvate was present, it reacted with H_2_O_2_ rapidly (**Fig. 4AB**). Consistent with these observations prior to the development of BSK-II medium, extracellular catalase addition was noted to promote the growth of *B. hermsii*, a related spirochete [79].

For *B. burgdorferi*, H_2_O_2_ caused DNA damage since mutants deficient in the NER or MMR DNA repair pathways exhibited enhanced sensitivity to H_2_O_2_ killing. Pyruvate fully protected three wild-type strains and both DNA repair mutants (Δ*uvrB* and Δ*mutS*). Although the mechanism of H_2_O_2_ killing for *L. interrogans* was not examined in this study, the addition of pyruvate also protected two pathogenic strains from H_2_O_2_ despite the presence of catalase within these spirochetes. Moreover, it is apparent that *L. interrogans* are more sensitive to H_2_O_2_ generated by GOX treatment than *B. burgdorferi*. *B. burgdorferi* was capable of growing, although at a rate severely below their potential, in response to GOX treatment; however, the same concentration of GOX reduced *L. interrogans* cell density to undetectable levels (**Figs. 2CD and 5**). This difference may be due to the lack of iron in *B. burgdorferi* metabolism compared to *L. interrogans*, which encodes several anabolic and catabolic pathways that require iron [15], [80]. Not surprisingly, *Leptospira* spp. require iron for growth [81]. Thus, *B. burgdorferi* is inherently more resistant to H_2_O_2_ than *L. interrogans*.

Pyruvate was capable of reducing both the intracellular and extracellular ROS produced by human neutrophils (**Fig. 6**). Our results suggest a possible bimodal pattern to inhibition of ROS by pyruvate; one that is more sensitive to pyruvate inhibition and another that is less sensitive. In the absence of additional data to identify and explain the cause for such a pattern of inhibition, we are reluctant to speculate on the ROS-generating factor(s) that may also be inhibited by pyruvate. Given the use of HRP in the luminol assay and the breadth of pyruvate in metabolism and immune cell regulation/activation, this is not a surprising result. Note that the ROS/neutrophil experiments conducted in this study were designed to specifically measure ROS production not killing. Preliminary data using the same MOI and conditions for measuring ROS did not reveal any difference in killing at the end of the experiment (data not shown). To completely address this issue in a rigorous manner, a detailed optimization of experiments will be needed.

It was expected that extracellular ROS produced by the plasma membrane localized NADPH oxidase complex would be quickly scavenged by extracellular pyruvate. Yet, intracellular ROS production was also diminished by pyruvate addition suggesting that pyruvate can efficiently enter neutrophil phagosomes. The extracellular addition of pyruvate to neutrophils is known to increase intracellular pyruvate concentrations [82]. Moreover, it is known that another ROS scavenger, NAC, reduces ROS and inhibits mitogen-activated protein kinase (MAPK) activation of the NADPH oxidase complex [75], [76]. Small molecular antioxidants may act by directly scavenging ROS or via regulating signal pathways dependent on ROS. NFκB and AP-1 are redox-dependent transcription factors that activate many antibacterial processes within immune cells and their functions are reduced by the antioxidant NAC [83], [84]. Furthermore, pyruvate reduced the ROS production in response to TLR2 activation by SOZ in human neutrophils. The host inflammatory response to *B. burgdorferi* lipoproteins requires TLR2 [85]. Moreover, upon interaction with host monocytes *B. burgdorferi* induces expression of host TLR2 and enters monocytes through a TLR2-dependent mechanism [86]. Because pyruvate reduces the ROS production by human neutrophils in response to both SOZ and *B. burgdorferi* it suggests that TLR2 expression or function may be perturbed. Additional work is needed to elucidate the precise mechanism of the role of pyruvate upon TL2-mediated inflammation in response to stimulus. Because pyruvate directly scavenges H_2_O_2_, a signal that activates host transcription factors NFκB and AP-1, pyruvate could reduce the activation of antibacterial pathways and protect pathogens against H_2_O_2_ damage.

Monocytes and neutrophils are competent to kill *B. burgdorferi in vitro* and *in vivo* [17], [72], [86], [87]. Despite the sensitivity of *B. burgdorferi* to ROS and RNS *in vitro*, there are no differences in *B. burgdorferi* burden within wild-type mice compared to mice lacking either the phagocytic NADPH oxidase [88] or the inducible nitric oxide synthase (iNOS) [89]. Because *B. burgdorferi* exploits salivary proteins in the tick vector for protection against ROS and host complement [4]–[8], it is likely that *B. burgdorferi* exploits additional factors to allow persistent infection. Pyruvate may be a factor within the host that *B. burgdorferi* utilizes for protection against ROS. Pyruvate can also scavenge RNS generated by the host. Peroxynitrite is a potent antimicrobial compound generated by the reaction between superoxide and nitric oxide and pyruvate scavenges peroxynitrite through a non-enzymatic reaction [90]. Similar to H_2_O_2_, peroxynitirite has been implicated in NFκB and AP-1 activation within neutrophils [91], [92]. Although the role of peroxynitrite in the host response against *B. burgdorferi* or *L. interrogans* is unknown, peroxynitrite is implicated in the host defense against bacterial pathogens [93], [94]. Thus, host pyruvate may scavenge H_2_O_2_ and additional reactive compounds during infection that modulate the host response to *B. burgdorferi* or *L. interrogans*.

Pyruvate is a ubiquitous metabolite. In human blood, pyruvate is typically within a range of ≈50 to 300 µM, but is subject to significant flux [63]–[67]. The concentration of pyruvate in blood and tissues colonized by *B. burgdorferi* during infection of the murine model of Lyme disease is currently unknown. In the guinea pig heart, pyruvate inhibits the ROS formation and cardiac NADH oxidase activity during ischemia/reperfusion events [95]. In the clinical setting, either bacterial-initiated sepsis or febrile bacterial infection increases blood pyruvate concentrations [96], [97]. During spirochete infection, the blood pyruvate concentration of laboratory rats infected with the relapsing fever spirochete, *B. recurrentis*, increase 3-fold during the peak of spirochetemia [98]. However, the physiological relevance and mechanism for this increase is unknown.

In conclusion, this work shows that *B. burgdorferi* and *L. interrogans* are sensitive to H_2_O_2_ killing. This work clarifies the discordant data from separate groups regarding sensitivity of *B. burgdorferi* to H_2_O_2_ killing, and further demonstrated the critical role of pyruvate in protection from H_2_O_2_ damage for both pathogenic spirochetes *B. burgdorferi* and *L. interrogans*. Finally, this work implies that extracellular pathogens may exploit pyruvate in host blood and tissue for protection against H_2_O_2_.
